# The Diagnostic Utility of Biochemical Markers and Intestinal Ultrasound Compared with Endoscopy in Patients with Crohn’s Disease and Ulcerative Colitis: A Systemic Review and Meta-Analysis

**DOI:** 10.3390/jcm13113030

**Published:** 2024-05-21

**Authors:** David Huynh, Denis Rubtsov, Debapama Basu, Myat Myat Khaing

**Affiliations:** The Prince Charles Hospital, Brisbane 4032, Australia; denis.rubtsov@health.qld.gov.au (D.R.); debapama.basu@health.qld.gov.au (D.B.); myatmyat.khaing@health.qld.gov.au (M.M.K.)

**Keywords:** C-reactive protein, erythrocyte sedimentation rate, ulcerative colitis, Crohn’s disease, inflammatory bowel disease, intestinal ultrasonography, endoscopy, diagnostic accuracy and biochemical markers

## Abstract

**Background:** Inflammatory bowel disease (IBD) consists of Crohn’s disease (CD) and Ulcerative colitis (UC). The main goal of treatment is to obtain mucosal healing via endoscopy. More recently, intestinal ultrasounds, along with biochemical markers, have been increasingly popular as point-of-care testing to monitor treatment response. This systemic review and meta-analysis aimed to assess the diagnostic test performance of ultrasonography and biochemical markers (C-reactive protein and fecal calprotectin) compared with endoscopy for detecting inflammation in IBD. **Methods**: A comprehensive literature search was conducted using PubMed Medline, EMBASE, ScienceDirect, and CINAHL from 1 January 2018 to 1 January 2024. The included studies were prospective and retrospective observational studies, clinical trials, and cross-sectional studies investigating the diagnostic sensitivity and specificity of ultrasonography, biochemical markers, and endoscopy. Studies were selected based on the Preferred Reporting Items for Systematic Review and Meta-analysis Statement (PRISMA). **Results:** Of the 1035 studies retrieved, 16 met the inclusion criteria, and most of the included studies were prospective observational studies. Diagnostic test accuracy was conducted, and the pooled sensitivity and specificity of all the studies revealed that ultrasonography has the highest pooled sensitivity, at 85% (95% CI, 78 to 91%), and specificity, at 92% (95% CI, 86 to 96%), as compared with biochemical markers and endoscopy. More specifically, biochemical markers had a pooled sensitivity and specificity of 85% (95% CI, 81 to 87%) and 61% (95% CI, 58 to 64%), respectively, and endoscopy had 60% (95% CI, 52 to 68%) and 82% (95% CI, 76 to 87%), respectively. However, the results also show substantial heterogeneity in the studies because of various populations, protocols, and outcomes in the studies included. This was especially noted in the assessment of biochemical markers, in which a metaregression was performed showing a nonsignificant *p*-value of 0.8856 for the coefficient. **Conclusions:** IUS was found to have the highest pooled sensitivity and specificity of all the included studies for diagnosing inflammation in patients with CD and UC, and this, coupled with biochemical markers, can improve diagnostic utility.

## 1. Introduction

Crohn’s disease (CD) and ulcerative colitis (UC) are the primary subtypes of inflammatory bowel disease (IBD), a chronic inflammatory condition affecting the gastrointestinal (GI) tract. Over recent years, there has been a notable increase in the global incidence of IBD [1,2]. The primary therapeutic goal in managing IBD is to achieve and sustain clinical remission. Integral to this objective is the attainment of mucosal healing, as confirmed through endoscopic evaluation, which correlates with reduced rates of hospitalization, bowel surgery, and improved overall prognosis [3,4].

While endoscopy remains the gold standard for assessing mucosal healing [5], biomarkers have emerged as valuable surrogates for inflammation and play a pivotal role in the comprehensive evaluation of patients with IBD. Among these biomarkers, C-reactive protein (CRP) and fecal calprotectin (FCP) are prominently utilized. CRP, an acute-phase protein primarily synthesized in response to interleukin (IL)-6 stimulation, serves as a marker of systemic inflammation, including in IBD [6,7]. Its utility lies in its ability to monitor disease activity and treatment response, although its reliability diminishes in cases of lower disease activity [8].

In contrast, calprotectin, a calcium-binding complex found predominantly in intestinal epithelial cells, offers a more specific assessment of intestinal inflammation. FCP reflects localized inflammation within the intestinal tract and boasts high stability, remaining unaffected by fecal collection methods due to the uniformity of fecal samples [9].

Moreover, the increasing adoption of intestinal ultrasound (IUS) in assessing IBD activity is attributed to its noninvasive nature and cost efficiency. IUS enables the identification of intestinal lesions, analysis of intestinal layers for inflammation (notably bowel wall thickness), visualization of mesenteric inflammation (MI), and detection of complications such as stenosis, dilatation, abscess, and fistulae [10,11]. Notably, the sensitivity of IUS can be enhanced when used in conjunction with CRP and FCP.

Although extensive literature exists on the individual roles of endoscopy, biomarkers, and ultrasonography in evaluating CD and UC, there remains a paucity of studies comparing their diagnostic performance comprehensively. Therefore, this meta-analysis aims to elucidate and compare the diagnostic efficacy of ultrasonography and biochemical markers (CRP and FCP) with endoscopy in detecting inflammation in patients with CD and UC.

## 2. Methods

A systematic review and meta-analysis were undertaken in accordance with the guidelines established by the Preferred Reporting Items for Systematic Reviews and Meta-analyses (PRISMA) for assessing diagnostic test accuracy [12]. Additionally, adherence to the Cochrane analytical methods for Diagnostic Test Accuracy studies was observed to ensure a comprehensive examination of the sensitivity and specificity of three distinct tests [13]. This systemic review was registered with PROSPERO (CRD42024546149).

1. Search Strategy

A thorough literature search was conducted across multiple databases, including PubMed Medline, CINAHL, EMBASE, and Science Direct, employing a comprehensive set of keywords encompassing various aspects of “Crohn’s disease”, “ulcerative colitis”, and “inflammatory bowel disease”, coupled with diagnostic modalities such as “ultrasonography”, “endoscopy”, and “biochemical markers”. These keywords were systematically combined into search strings, and a publication date range spanning from 1 January 2018 to 1 January 2024 was applied. The literature search was performed by DH. Additionally, the search was confined to studies involving human subjects, predominantly adults aged 18 years and above, with exclusion criteria excluding studies involving children. English language publications were prioritized, while no geographical restrictions were imposed, ensuring the inclusion of studies from diverse regions.

2. Inclusion and Exclusion

In the current review, comprehensive eligibility criteria were used to select the most relevant studies in the meta-analysis. The inclusion criteria were as follows:

- Adult population with CD and UC.
- Index tests, including ultrasound, endoscopy, and biochemical markers (Calprotectin and C-reactive protein).
- Comparator test endoscopy and other tests.
- Outcomes include sensitivity and specificity of index tests active inflammation and remissions.
- Study design involving retrospective and prospective observational studies, as well as clinical trials.

The exclusion criteria were as follows:

- Studies with child populations.
- Studies with index tests other than the target tests and biomarkers.
- Studies that have not compared the index test with comparator tests.
- Studies that lacked details for sensitivity and specificity.
- Case reports and all types of review articles were excluded.

3. Data Extraction and Quality Assessment

Upon careful selection of studies for inclusion in the review, pertinent data were extracted, encompassing study characteristics such as author names, publication years, and regions, along with details on the study population, methodology, index and comparator tests, and diagnostic outcomes. The Quality Assessment of Diagnostic Accuracy Studies (QUADAS-2) tool was utilized to appraise the quality of the included studies, renowned for its comprehensive evaluation across four domains: patient selection, index test, reference test, and patient flow. All included studies underwent independent quality assessment by two reviewers, DH and DR, with any discrepancies rigorously cross-checked for resolution. These studies were then discussed and reviewed by MMK to confirm whether they were suitable for inclusion. In addition, any discrepancies were resolved in consultation with MMK.

4. Outcome Variables

The present study focused on the outcome variable of the accurate detection of inflammation in patients with CD and UC using ultrasonography, biochemical markers (CRP and FCP), and the endoscopy method.

5. Data Synthesis and Statistical Analysis

The diagnostic performance data from all studies were synthesized to assess sensitivity and specificity collectively. Utilizing forest plots, estimates of diagnostic performance for the three tests were graphically represented, illustrating observed sensitivities and specificities. Additionally, summary receiver operating characteristic (SROC) curves were generated to depict individual study results. Heterogeneity within the pooled sensitivities and specificities across the three tests was assessed using the Cochrane Q test (*p* < 0.05) and Higgins I^2^ statistics, with an I^2^ value exceeding 50% indicating significant heterogeneity. This analytical process was conducted using Revman Review Manager (version 5.4.1) and MetaDiSc (Version 1.4), as per established methods [14].

## 3. Results

1. Literature Search

**Figure 1:**
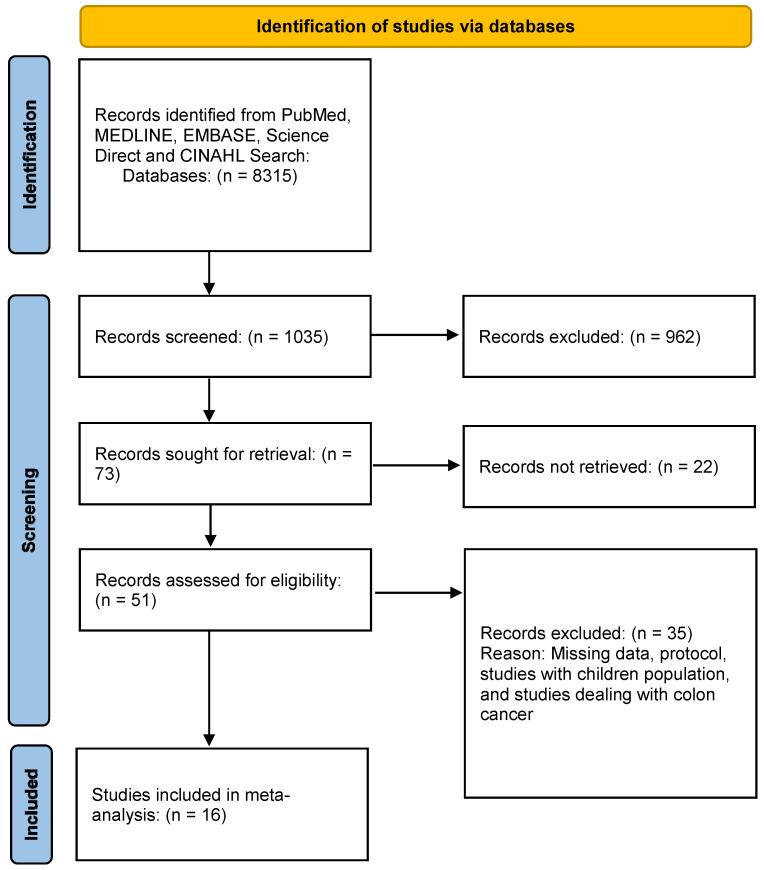
PRISMA flow diagram of identification of studies on diagnostic performance of ultrasonography, endoscopy, and biochemical markers.

The initial literature search across databases yielded 1035 studies relevant to CD, UC, and various diagnostic tests. These retrieved records underwent screening, applying predetermined filters aligned with the eligibility criteria. Following this process, the number of relevant studies was refined to 51 articles. Subsequently, the full-text PDFs of these articles were acquired and scrutinized for eligibility, resulting in the inclusion of only 16 studies that met the predefined criteria for further meta-analysis. Please see Figure 1, summarizing the PRISMA flow diagram of identified studies.

2. Characteristics of Included Studies

The main characteristics of all the 16 included studies are provided in Table 1. Out of the total 16 studies, 12 studies were prospective observational studies [15,16,17,18,19,20,21,22,23,24,25], 2 were retrospective studies [26,27], 1 was cross-sectional [28], and 1 was a multicenter randomized controlled trial [29]. There were 382 patients in studies investigating the diagnostic accuracy of endoscopy [17,25,26,29], 1818 patients in the studies investigating the diagnostic accuracy of biochemical markers [15,16,18,19,21,23,24,27,28], and 146 patients in the studies investigating the diagnostic accuracy of ultrasonography [20,22,30].

3. Quality of Studies

**Figure 2:**
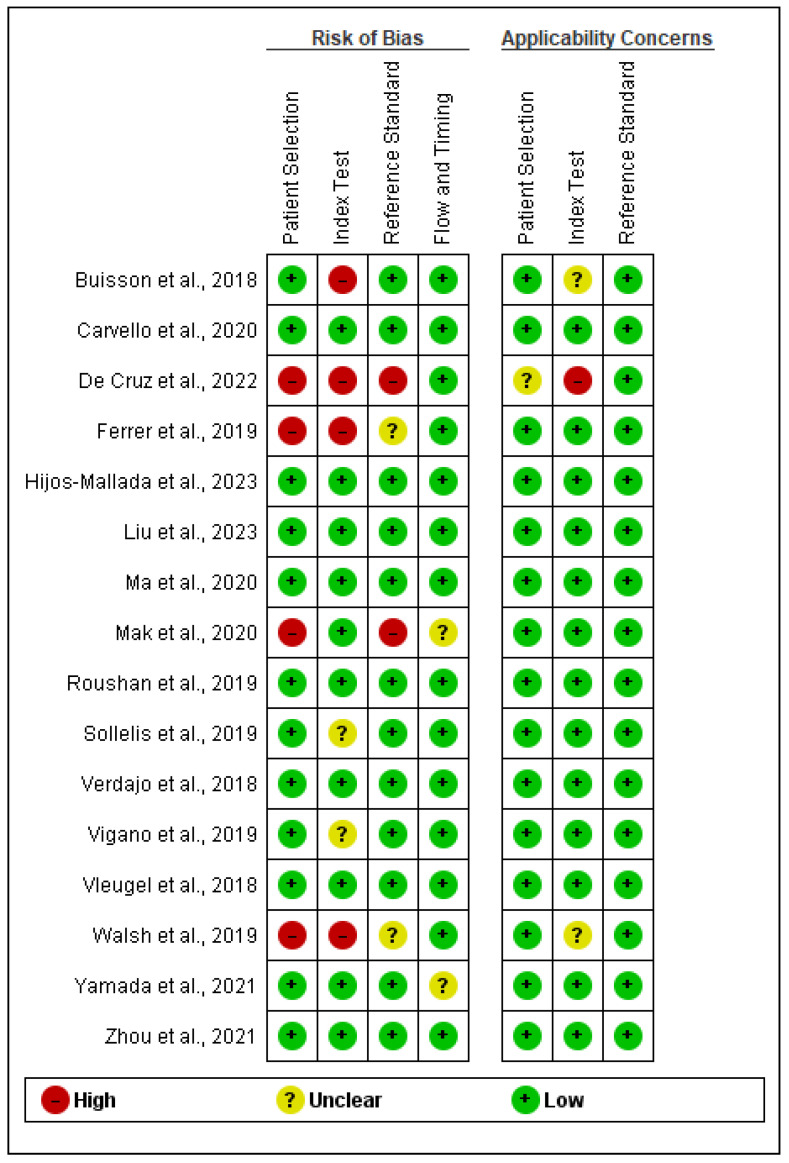
Methodological quality of included studies [15,16,17,18,19,20,21,22,23,24,25,26,27,28,29,30].

**Figure 3:**
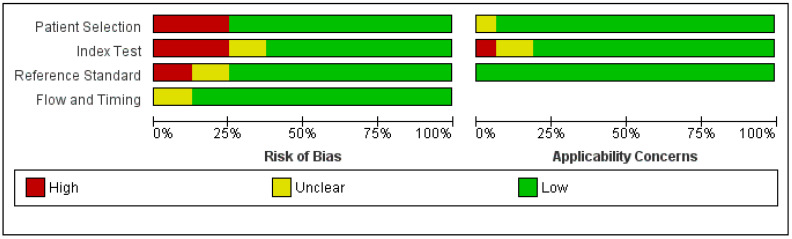
Overall quality of the studies.

The quality of the studies underwent assessment utilizing the QUADAS-2 criteria, the findings of which are encapsulated in Figure 2 and Figure 3. This was performed in RevMan software (version 5.4.1), which offers a built-in QUADAS-2 tool for the evaluation of the risk of bias and applicability of diagnostic accuracy studies. In the RevMan software, there are options for QUADAS-2 domains with different signaling questions. For instance, under the risk of bias domain, there are five signaling questions, such as patient sampling, randomization of enrolled patients, avoiding case-control design, avoiding any inappropriate exclusions, and whether there is any risk of bias during the selection of patients. These questions are answered as high risk, unclear risk, and low risk [31].

Several notable issues emerged regarding patient selection across the included studies. Firstly, four studies raised concerns pertaining to patient selection. This domain within the QUADAS-2 framework scrutinizes the representativeness of the study cohort. Notably, one study failed to furnish details regarding participant and researcher blinding, thus engendering a risk of selection bias [15]. Another study restricted participant recruitment to a singular center, potentially constraining the generalizability of its findings [16]. Additionally, the inadvertent exclusion of certain patient subgroups was noted in one study, likely compromising the representativeness of the sample and consequently the generalizability of the results [19]. Furthermore, instances of selection bias and heterogeneity within the study population were observed, thereby impeding the accurate interpretation of the findings [22].

According to the QUADAS-2 criteria, comprehensive description and execution of the index test are imperative. Regrettably, two studies lacked clarity regarding the index tests employed [21,28], partly due to the multitude of tests conducted. Moreover, four studies exhibited a high risk of bias in their index test domain. Notably, one study utilized the POCER index test, which has been deemed unreliable and is based on a POCER study [15]. In another study, the Harvey Index and Walmsley Index tests were utilized for CD and UC assessment, respectively, instead of the more widely accepted CDEIS and UCEIS indices [16].

Overall, while concerns regarding the applicability of the reviewed studies are relatively limited, notable issues related to patient selection and index test utilization were identified. Nonetheless, these concerns could be mitigated through the adoption of context-specific interpretations for these studies.

4. Diagnostic Performance of Ultrasonography

**Figure 4:**
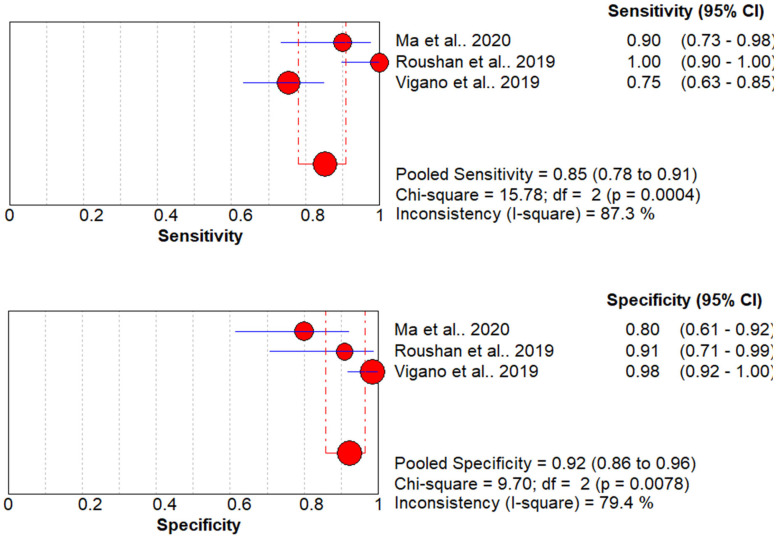
Coupled forest plots of overall pooled sensitivity and specificity for diagnosis of UC and CD using ultrasonography [20,22,30].

The diagnostic performance of ultrasonography was reported in three studies with 146 patients [20,22,30]. The pooled sensitivity and specificity of these studies were 85% (95% CI, 78 to 91%) and 92% (95% CI, 86 to 96%), respectively. The Higgins I^2^ statistics were found to be 87.3% and 79.4% in sensitivity and specificity, respectively. This shows that there is significant heterogeneity in the sensitivity, while lower heterogeneity was observed in the specificity (Figure 4). The 95% confidence interval (CI) for the I^2^ statistics ranges from 65.6% to 95.6%.

5. Diagnostic Performance of Biochemical Markers

**Figure 5:**
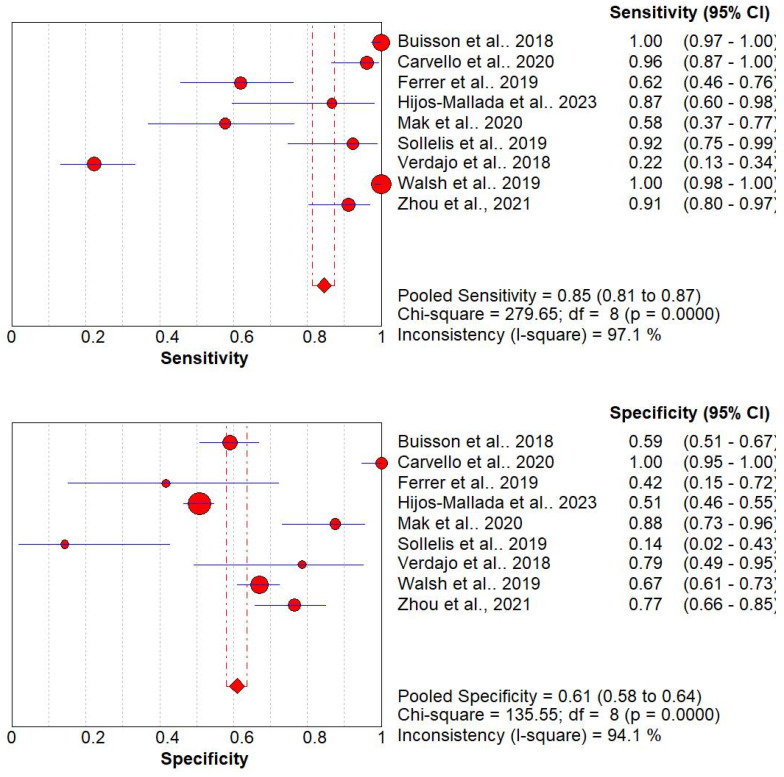
Coupled forest plots of overall pooled sensitivity and specificity for diagnosis of UC and CD using biochemical markers (CRP and FCP) [15,16,18,19,21,23,24,27,28].

The diagnostic performance of biochemical markers was reported in nine studies with 1818 patients [15,16,18,19,21,23,24,27,28]. The pooled sensitivity and specificity of these studies were 85% (95% CI, 81 to 87%) and 61% (95% CI, 58 to 64%), respectively. The Higgins I^2^ statistics were found to be 97.1% and 94.1% in sensitivity and specificity, respectively. This shows that there is significant heterogeneity in the sensitivity and specificity (Figure 5). Due to significant heterogeneity, a metaregression was performed to explore potential sources of heterogeneity. See Table 2 for the metaregression of the nine included studies. The coefficient of this predictor variable is 0.071 with a nonsignificant *p*-value of 0.8856. Furthermore, the estimate of Tau-squared (1.0163) represents the between-study variance, indicating the variability in effect sizes across studies after accounting for sampling error.

Tau-squared estimate = 1.0163 (convergence is achieved after six iterations). Cte is a constant. g represents the covariate, which in the current case is the type of condition (Crohn’s disease, ulcerative colitis, or both).

6. Diagnostic Performance of Endoscopy

**Figure 6:**
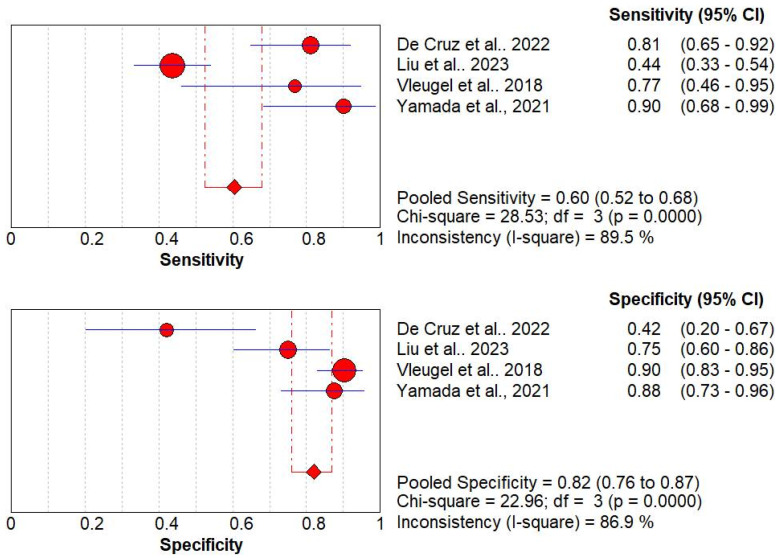
Coupled forest plots of overall pooled sensitivity and specificity for diagnosis of UC and CD using endoscopy [17,25,26,29].

The diagnostic performance of endoscopy was reported in four studies with 382 patients [17,25,26,29]. The pooled sensitivity and specificity of these studies were 60% (95% CI, 52 to 68%) and 82% (95% CI, 76 to 87%), respectively. The Higgins I^2^ statistics were found to be 89.5% and 86.9% in sensitivity and specificity, respectively. This again shows that there is a significant heterogeneity in the sensitivity and specificity (Figure 6).

**Figure 7:**
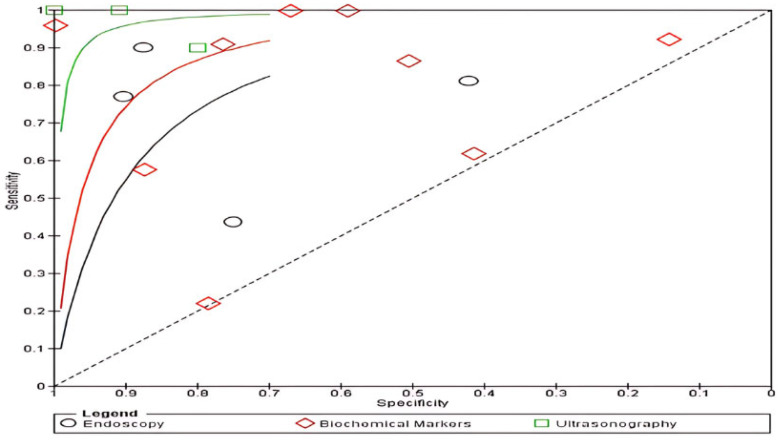
Summary receiver operating characteristic (SROC) curve of diagnostic performances of all three tests for diagnosing inflammation in CD and UC.

The summary receiver operating characteristic (SROC) curve for diagnostic performances is provided in Figure 7. It shows that the sensitivity and specificity were reported to be highest in ultrasonography.

## 4. Discussion

While endoscopies with histological samples are still the gold standard in evaluating IBD flares and treatment monitoring, IUS has garnered recognition as a noninvasive, cost-effective, and efficient technique for monitoring patients with IBD [11,32]. This is especially true as there are limitations on how quickly endoscopy can occur due to logistical reasons, such as patient preference, timing, availability, and fasting status. The utilization of IUS has been proposed as a valuable means for screening IBD, particularly in evaluating the anatomical extent of CD at initial diagnosis [33,34]. A previous meta-analysis investigating the diagnostic accuracy of IUS in identifying active CD reported combined sensitivity and specificity rates of 88% and 97%, respectively. Furthermore, this study indicated that ultrasound could discern both CD and UC with sensitivity rates of 86% to 89% and specificity rates of 95% to 97%, respectively [35,36]. In terms of biochemical markers, FCP holds significance in accurately distinguishing between active and inactive disease states, particularly in patients undergoing IBD treatment. Similarly, CRP emerges as a highly sensitive surrogate serologic marker of inflammation in adults, surpassing other markers. Notably, its sensitivity in distinguishing CD from irritable bowel syndrome ranges from 70% to 100% and from 50% to 60% for UC [37]. Active CD is associated with elevated CRP levels compared with UC, suggesting a potential differentiating factor [8,38].

While previous meta-analyses only focused on IUS accuracy, the present meta-analysis is the first systemic review and meta-analysis to combine and compare both the diagnostic tests of the rising popularity of IUS with biochemical markers (CRP and FCP) and the gold standard of endoscopies among patients with IBD. Our findings revealed a significantly higher sensitivity of 85% for ultrasonography compared with 60% for endoscopy and 85% for biochemical markers. Similarly, ultrasonography exhibited superior specificity at 92%, contrasted with 61% for biochemical markers and 82% for endoscopy. These results underscore the efficacy of ultrasonography in accurately diagnosing inflammation and monitoring recovery in CD and UC patients, noting that significant heterogeneity was observed in the pooled sensitivity and specificity for ultrasonography, biochemical markers, and endoscopy.

The diagnostic performance of biochemical markers was specifically evaluated, revealing a sensitivity of 85% (95% CI, 81 to 87%) and a specificity of 61% (95% CI, 58 to 64%). Our review underscores a notably robust sensitivity of biochemical markers in diagnosing IBD, consistent with previously documented ranges [32]. However, it is noteworthy that the aggregated specificity of these markers was found to be lower than previously reported, although this characteristic may facilitate the initial detection of inflammation due to the test’s high sensitivity. Additionally, endoscopic procedures demonstrated a pooled sensitivity and specificity of 60% (95% CI, 52 to 68%) and 82% (95% CI, 76 to 87%), respectively. Of note, the sensitivity of 60% in detecting inflammation suggests that microscopic disease activity may elude detection via endoscopy [39,40]. Conversely, the higher specificity of 82% is valuable in excluding inflammation in patients with inflammatory bowel disease (IBD). These results are also comparable to other published data.

These findings suggest that the combined use of IUS and biochemical markers could serve as potent assessment tools in clinical settings for gastroenterologists, particularly when ultrasonography resources are readily accessible. While the sensitivity and specificity of IUS in detecting inflammation are relatively high, it is imperative to acknowledge that IUS alone cannot ascertain mucosal healing. Nonetheless, its utilization can be invaluable in assessing treatment response in acute hospitalized settings and outpatient clinic settings, as well as identifying potential disease flares, thereby mitigating the need for invasive procedures. It is important to emphasize that patients may still require endoscopies to confirm remission.

Several limitations are notable in our review. Firstly, the number of studies included for diagnostic accuracy assessment was limited. Secondly, studies exhibited notable heterogeneity, necessitating metaregression analysis to explore potential sources. We believe that the high heterogeneity is related to differences in study populations, methodologies, and different outcomes of each study, such as different biochemical markers. Thirdly, heterogeneous reference standards were employed in the included studies, including endoscopy, histological findings, and other biomarkers. These limitations necessitate further investigation in the future to offer a comprehensive review of the diagnostic performance of these tests. Lastly, the generalizability may be impacted, as the included studies and data did not encapsulate the Southeast Asian population, which could potentially affect the utility of IUS and biochemical markers.

Moreover, we recognize the significance of delving into potential sources of heterogeneity to comprehensively grasp the variations in sensitivity and specificity estimates across studies. While our investigation did not directly delve into the factors contributing to this heterogeneity, we acknowledge that numerous variables could potentially impact the efficacy of biochemical markers. These may encompass differences in patient demographics, study methodologies, and geographical disparities, which although not explicitly addressed in our analysis, could significantly influence observed heterogeneity.

Additionally, we acknowledge the diversity of ultrasound techniques evaluated in the three studies included in our analysis. Such variations have introduced heterogeneity, thereby constraining the generalizability of our findings. The assorted ultrasound methodologies may affect diagnostic accuracy and reliability, as evidenced by the I^2^ heterogeneity displayed in the accompanying figure. Nevertheless, the adoption of specific ultrasound techniques in clinical settings demands meticulous consideration of various factors, including operator proficiency, equipment accessibility, patient comfort, and cost-effectiveness. To confront these challenges and enhance the clinical utility of ultrasound, there is an urgent imperative to establish standardized ultrasound protocols tailored to specific clinical contexts. Such standardization endeavors could entail the formulation of consensus guidelines or recommendations delineating optimal practices for conducting and interpreting ultrasound examinations in gastrointestinal imaging. These protocols should encompass considerations such as patient preparation, imaging acquisition parameters, interpretation criteria, and quality assurance measures. Perhaps, in the future, another systematic review and meta-analysis can be performed once IUS becomes a more standardized practice.

## 5. Conclusions

The diagnostic test sensitivity and specificity of IUS in detecting inflammation among patients with CD and UC were observed to surpass those of biochemical markers and endoscopy. These results underscore the efficiency of ultrasonography in precisely assessing inflammation, particularly when complemented by biochemical markers. Such combined approaches offer valuable utility as rapid and accessible point-of-care assessments for identifying flareups or evaluating treatment responses in contrast to relying solely on endoscopy, which may delay decision making due to timing and logistics, such as fasting status and availability. This also could greatly improve the efficiency of outpatient gastroenterology services. Ultimately, however, endoscopy is still required to assess mucosal healing.

## Figures and Tables

**Table 1:** Characteristics of Included Studies.

Authors’ Name	Publication Year	Publication Region	Sample Size	Study Population	Study Methodology	Index Test	Comparator Test	Diagnostic Outcomes
Vleugels et al. [27]	2018	UK and The Netherlands	210	Patients with long-standing UC	Multicenter, randomized controlled trials	Endoscopic trimodal imaging	Histopathology	ETMI has a diagnostic accuracy of 88.8%
Walsh et al. [13]	2019	UK	39	Patients with UC	Prospective study	UCEIS for FCP	Histological (Nancy)	Weak correlation between FCP and UC activity
Mak et al. [14]	2020	Hong Kong	113	Patients diagnosed with UC and CD	Prospective study	FCP and blood markers	Endoscopy	Combination of both fecal markers and blood indices provide better diagnostic performance as compared with fecal biomarkers alone
Ferrrer et al. [15]	2019	Spain	53	Patients with established diagnosis of UC and CD	Prospective study	Serum calprotectin	Endoscopic findings	Serum calprotectin provides accurate assessment of inflammatory activity
Ma et al. [16]	2020	China	30	Inpatients with CD and UC	Prospective study	Multimodal ultrasound	Endoscopy	Multimodal ultrasound produced detailed clinical values
De Cruz et al. [17]	2022	Australia	85	Patients with CD	Prospective randomized controlled trial	Endoscopy		POCER postoperative index provide in-depth details of patients who need intensive therapy
Hijos-Mallada et al. [18]	2023	Spain	571	patients referred for colonoscopy	Prospective observational Study	FCP	Other biomarkers	Point of care FCP prevent unnecessary colonoscopies in high-risk patients
Liu et al. [25]	2023	China	166	Patients with confirmed UC	Retrospective observational study	TIGER scores	UCEIS	TIGER scores were found to be superior compared with UCEIS
Yamada et al. [19]	2021	Japan	22	Patients with CD	Prospective study	PillCam colon capsule endoscopy (PCCE-2)	Endoscopy	PCCE-2 has a better diagnostic yield compared with simple endoscopy
Buisson et al. [20]	2018	France	86	Patients with IBD	Prospective observational Study	Fecal markers	Endoscopy	Fecal matrix metalloprotease with better markers
Zhou et al. [24]	2021	China	112	Patients with CD	Retrospective observational study	CRP	Other biomarkers	CRP is an appropriate biomarker for mucosal healing in CD
Verdejo et al. [26]	2018	Spain	86	Patients with CD	Cross-sectional, observational, multicenter cohort study	CRP	FCP	CRP is better predictor for CD as compared with FCP
Carvello et al. [21]	2020	Italy	345	Patients with CD	Prospective study	C-reactive protein	Other biomarkers	CRP trends predict the early discharge of patients with CD
Sollelis et al. [22]	2019	France	40	Patients with CD	Prospective study	C-reactive protein	Other biomarkers	Combined monitoring of biomarkers provides better prediction about outcomes in CD patients
Vigano et al. [28]	2019	Italy	65	Patients with CD	Prospective study	Intraoperative ultrasonography (IOUS)	Endoscopy	IOUS is feasible for patients with CD
Roushan et al. [23]	2019	Iran	70	Patients with CD and UC	Prospective, single-blinded study	Endoscopic ultrasonography	Endoscopy	EUS is efficient in diagnostic accuracy of UC and CD

Acronyms: FCP—fecal calprotectin; CD—Crohn’s disease; UC—ulcerative colitis; CRP—C-reactive protein; UCEIS—ulcerative colitis endoscopic index score; IOUS—intraoperative ultrasonography; TIGER—Toronto inflammatory bowel disease global endoscopic reporting; ETMI—endoscopic trimodal imaging.

**Table 2:** Metaregression of Nine Studies of Biochemical Markers.

Predictor Variables	Coefficient	Std. Error	*p*-Value	RDOR	95% CI
Cte	2.148	0.5482	0.0078	-	-
g	0.071	0.4744	0.8856	1.07	(0.34; 3.34)

## Data Availability

Data sharing is not applicable to this article as no datasets were generated or analyzed during the current study.
